# Interprofessional day – an educational event for medicine and the allied health professions at the University of Lübeck

**DOI:** 10.3205/zma001816

**Published:** 2026-02-17

**Authors:** Annemarie Minow, Janina Barth, Jürgen Westermann

**Affiliations:** 1 Universität zu Lübeck, Referat Studium und Lehre Humanmedizin, Lübeck, Germany; 2 Universität zu Lübeck, Institut für Anatomie, Lübeck, Germany

**Keywords:** health professions, curriculum development, interprofessional education, interprofessional relations, teaching methods

## Abstract

**What is the context or background of the project?:**

“Interprofessional day” was called into being to address the growing importance of interprofessional collaboration in healthcare. Participants are all students in their second semester of study in medicine, nursing, physiotherapy, midwifery, occupational/speech therapy, health and healthcare sciences, and applied nursing science at the University of Lübeck (UzL). This full-day event is a permanent fixture in the curricula of these degree programs.

**Why was the project started?:**

Since 2014 a number of healthcare degree programs have been created at UzL opening up new opportunities for interprofessional teaching and learning. Interprofessional day offers students the chance to strengthen their sense of belonging to the university's medical department, break down social barriers and become more consciously aware of the roles in interprofessional collaboration.

**How was the project carried out?:**

In 2024 this included a main session to open and close the event plus a group phase during which the students attended two workshops.

**How was the project evaluated?:**

The evaluation in 2024 was done online using a self-devised questionnaire which was completed by 160 out of 260 students. The evaluation of the interprofessional day was overall positive. The workshop on health knowledge insights received the highest ratings. The question about which aspects of the event were most successful elicited responses identifying the mixed groups and the workshops, among other aspects. In their responses to the query about what should be changed, the students mentioned, among other things, the length of the opening session and the weekday on which the event was held.

**Final overall assessment and outlook:**

This project contributes to the interaction and exchange between students pursuing different professions, the breakdown of social barriers and emphasizes the importance of teamwork to patient care. This event will continue with revisions to its concept and the possibility of including trainees in the allied health professions and students in the psychology degree program.

## Zusammenfassung

**Was ist der Kontext bzw. Hintergrund des Projekts?:**

Der Interprofessionelle Tag wurde initiiert, um die wachsende Bedeutung interprofessioneller Zusammenarbeit im Gesundheitswesen zu adressieren. Alle Studierenden des 2. Fachsemesters der Humanmedizin, Pflege, Physiotherapie, Hebammenwissenschaft, Ergotherapie/Logopädie, Gesundheits- und Versorgungswissenschaften und Angewandte Pflegewissenschaft der Universität zu Lübeck (UzL) nehmen teil. Die ganztägige Veranstaltung ist ein fester Bestandteil des Lehrplans der aufgeführten Studiengänge.

**Warum wurde das Projekt begonnen?:**

Seit 2014 sind an der UzL mehrere gesundheitsbezogene Studiengänge entstanden, die neue Möglichkeiten für interprofessionelles Lehren und Lernen eröffnen. Der Interprofessionelle Tag bietet den Studierenden die Gelegenheit, das Zugehörigkeitsgefühl zur Sektion Medizin zu stärken, soziale Barrieren abzubauen und die Rolle in der interprofessionellen Zusammenarbeit bewusster wahrzunehmen.

**Wie wird das Projekt durchgeführt?:**

Die Veranstaltung umfasste 2024 neben einer zentralen Auftakt- und Abschlussveranstaltung, eine Gruppenphase, in der die Studierenden zwei Workshops besuchten.

**Wie wird das Projekt evaluiert?:**

Die Evaluation erfolgte online im Jahr 2024 anhand eines selbstentwickelten Fragebogens, den 160 von 260 Studierenden beantworteten. Die Evaluation des Interprofessionellen Tags fiel insgesamt positiv aus. Die Gesundheitswissen Insights-Workshops wurden am besten bewertet. Bei der Frage, welche Aspekte der Veranstaltung gelungen waren, wurden u. a. die gemischten Gruppen und die Workshops genannt. Auf die Frage, was geändert werden soll, wurden u. a. die Länge der Auftaktveranstaltung und der Wochentag der Veranstaltung genannt.

**Abschließende Gesamteinschätzung und Ausblick:**

Das Projekt trägt zum Austausch zwischen Studierenden verschiedener Professionen sowie dem Abbau sozialer Barrieren bei und betont die Bedeutung von Teamarbeit für die Patient:innenversorgung. Das überarbeitete Konzept wird mit einer möglichen Ausweitung auf Auszubildende der Gesundheitsfachberufe und dem Studiengang Psychologie fortgeführt.

## 1. What is the context or background of the project?

With improved coordination, more efficient courses of therapy and a higher quality of care, well-functioning interprofessional collaboration is viewed as an essential part of patient-centered healthcare [1]. Already in 2010, the WHO called for such collaborative healthcare structures [2]. However, significant challenges are still faced in practice, such as an unclear assignment of roles between the professions and deficits in the ongoing communication of information. The decisive factors for sustainably anchoring interprofessional collaboration are support of open communication, shared decision-making and mutual respect, among others [3].

Interprofessional day was developed against the backdrop of the steadily growing importance of good interprofessional collaboration to optimal patient care [4], [5] and the addition of several new degree programs in the health sciences at the University of Lübeck (UzL).

Whereas the medical degree program has been an established course of study at UzL since 1964, other health-related degree programs have been steadily added since 2014 when a bachelor degree program in nursing was first offered. 2016 saw the addition of the bachelor degree program in physiotherapy, midwifery was added in 2017, and occupational/speech therapy in 2018. The master degree program in health and health sciences followed in 2019, and then the bachelor degree program in applied nursing science in 2022. All of these degree programs are housed in the Department of Medicine.

Research findings regarding student attitudes toward interprofessional collaboration point to an early inclusion of interprofessional education in the curricula [6], [7]. Furthermore, an interprofessional impetus can be directed at several degree programs to heighten students’ awareness of collaboration and their interprofessional identities and to prepare them for cooperation in healthcare [8].

Interprofessional day is an integrative project aimed at first-year students in the second semester of study in the department of medicine. It is also permanently integrated in the course schedules of all of the degree programs offered by the department of medicine.

## 2. Why was the project started?

As a result of establishing a number of degree programs in health science, all in the medical department, there is not only a new set of challenges, but now opportunities to bring people in different professional groups together while they are still pursuing their education. Among these opportunities is, in particular, the chance to gain an understanding of the tasks, functions and responsibilities of other health professions. The students can thus dispel stereotypes, practice interprofessional communication and establish shared values to guide such collaboration. This builds a valuable foundation for future professional practice. In addition, due to the institutional proximity of the degree programs on one campus, common teaching formats can be developed, which increases Lübeck’s attractiveness as a place to study the health professions. At the same time, challenges also arise from the heterogeneity of the degree programs; differing course schedules and academic prerequisites increase the complexity of coordinated planning and scheduling for the interprofessional courses. Especially challenging in terms of organization are finding suitable spaces and ensuring equal participation of all of the professions. Moreover, there is also the challenge of promoting a balanced representation of and mutual respect for all of the professional groups, even when the actual number of students is weighted unequally among the degree programs. An example of successful interprofessional learning on equal footing can be seen in the human dissection course offered at UzL [9]. Other multi- and inter-professional courses (e.g., lectures on orthopedics, surgery, internal medicine and gynecology; the module on interprofessional communication and care) are threaded longitudinally throughout the entire course of study at the department of medicine.

Interprofessional day is meant to unite all of the professions represented in the Department of Medicine (medicine, nursing, physiotherapy, midwifery, occupational/speech therapy, health and healthcare sciences, and applied nursing science) – the only program in Germany referred to as “the healthcare sciences under one roof” – and to communicate a shared understanding to the larger world. The event also contributes to developing and intensifying a sense of belonging to the department that goes beyond purely professional perspectives. The central purpose of this event for the students is to make them feel that they are members of the department's community and to encourage interprofessional interactions between them to help them recognize the personal relevance that interprofessional collaboration has for them. At the same time, it is also meant to break down social barriers, strengthen the ability to work in teams and develop a shared professional identity. Especially important is cooperative learning with and from each other. Interprofessional day serves as the first curricular link in this integrative learning process.

Originally organized as a campus rally with brief workshops and game-like challenges, Interprofessional day also served to showcase campus activities and services, such as university sports and the counseling services offered by the students’ union, to the second-semester students. At its inauguration in 2018, the event helped to present the new degree programs offered by the department of medicine and promote Lübeck as a place of higher learning to pursue an academic degree in the health sciences. Based on the evaluations up until then, students desired in-depth information about the field of health and information on the responsibilities and competencies of the professional groups. This then led to a revision of the learning objectives (LO).

The students who attend the event:

1. Get to know the students pursuing degrees in the other professions,
2. Become familiar with the other professions that can be studied in the separtment of medicine and how they link to their own profession,
3. Identify and reflect on their values and notions regarding interprofessional collaboration,
4. Work with other students to formulate a shared guiding principle for interprofessional collaboration,
5. Gain insight into another profession during a practical workshop.

## 3. How was the project carried out?

In its new rendition, interprofessional day consists of an opening session, two workshops entitled “100 minutes of team culture” (TC workshop) and “100 minutes of heath knowledge insights” (HKI workshop), and a closing session. The schedule for the interprofessional day held in 2024 is presented in table 1 (Tab. 1).

The opening session was comprised of a greeting and introduction to the event as well as a presentation on the competencies and scopes of responsibility for each of the participating professions. Information was gathered from the participants to answer the question: What do doctors/physiotherapists/midwives/etc. actually do? The students were instructed to list on a digital pinboard (padlet) what they thought belonged to the responsibilities and competencies of each profession. One or two representatives from each profession then took the stage and commented on this list, adding to it, for instance, the tasks or responsibilities that had not yet been mentioned and addressing the clichés they felt their professional group was constantly confronting. In the course of this, the students learned about the different professions and how they intersected with the profession they were pursuing (LO 1, LO 2). After this segment came some practical details about starting the group phase.

Prior to the event, the students had the opportunity to select a HKI workshop corresponding to their personal interests. The choice was between seven topics from the professions of medicine, physiotherapy, midwifery, occupational and speech therapy (e.g., talking reduces risk, violence in obstetrics, interprofessional collaboration for patients with dysphagia). Attention was paid during workshop registration so that as many of the degree programs as possible were represented in each group.

All of the participants attended a HKI workshop and a TC workshop during the group phase. The composition of the groups was based on the registrations for the HKI workshop. Half of the groups began with the HKI workshop, the other half with the TC workshop. Half an hour of time was planned between 10:00-12:10 to break the ice and get acquainted before starting the workshop.

The seven HKI workshops shared the goal of reinforcing interprofessionalism in the health sciences in that they offer the participants practical insights into the cooperation between the different health professions. The participants learn how important it is to understand and integrate the perspectives and expertise of the other disciplines in order to ensure holistic patient care. In doing this, the focus is placed on communication, shared decision-making and understanding the roles of and challenges faced by the other professional groups (LO 5).

Teachers from the degree programs moderated the TC workshops. The aim of the TC workshop is for participants to get to know each other, share their prior experiences with teamwork and explore common values to articulate a guiding principle for team culture (LO 3, LO 4). To get started during the TC workshop, the students are first asked to write down values for working together on cards that are then collected for group reflection and discussion. Following this, using the world café method, the following questions are discussed in small groups of 6-7 participants.

- In your opinion, which skills and competencies are important for working successfully in interprofessional teams?
- Recall previous group work/teamwork (e.g., school, internships). When did cooperation fail?
- What makes for good (interprofessional) collaboration? What is the outcome?

Based on the discoveries from the world café, the final part of the TC workshop asks the group participants to formulate a guiding statement on the topic of “What do I do so that we can work well together?” What the response to this should look like is left open.

**Figure 1:**
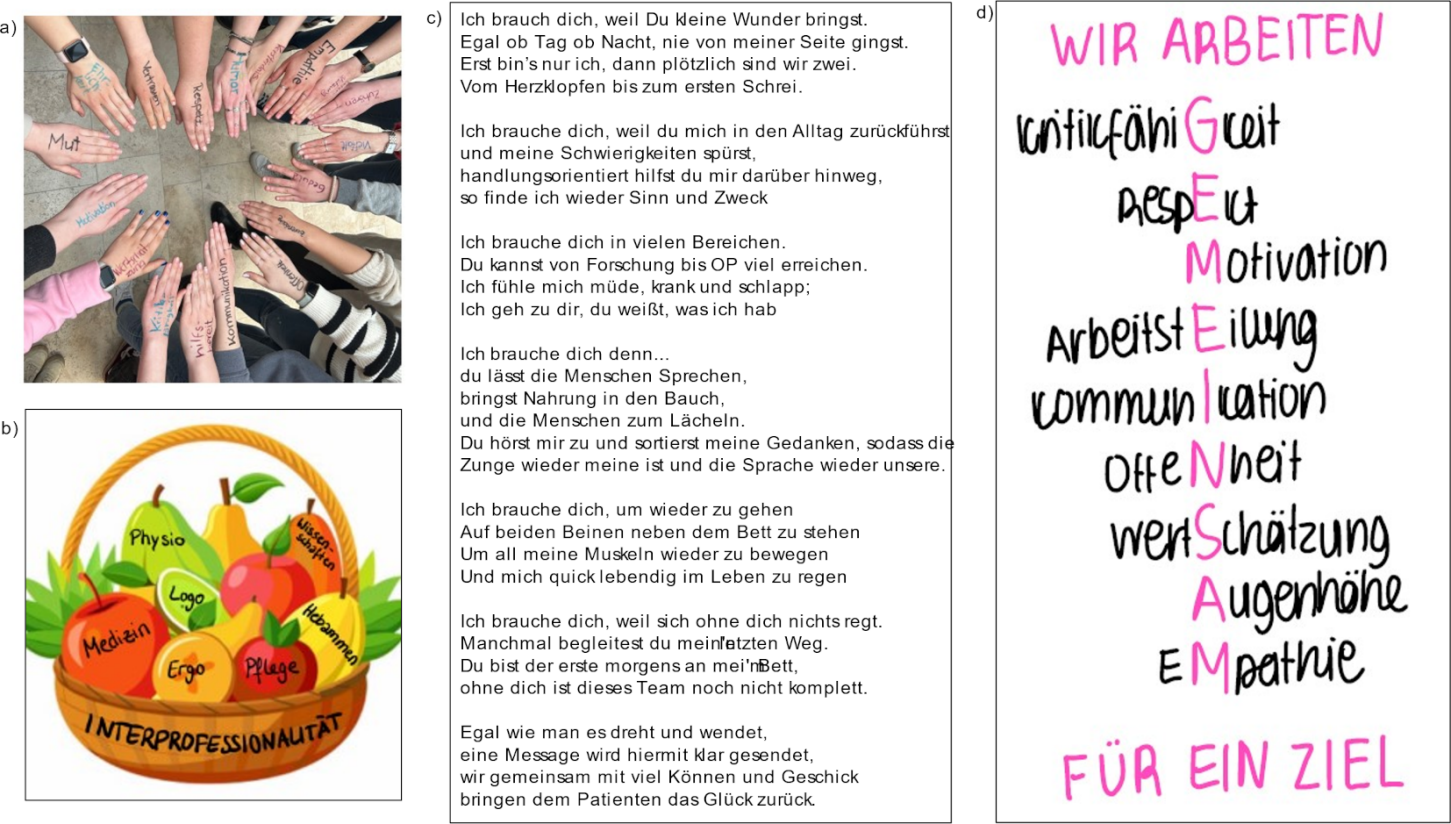
A selection of the work from the "100 Minutes of team culture" workshops (only in German) a) “Many hands, one good ending!” b) “One apple alone does not fill a fruit basket.” c) Poem on the professions (one stanza for each profession) d) Mission statement: “We work together to achieve one aim.” Vertical list of words: “Ability to take criticism, respect, motivation, division of labor, communication, openness, esteem, equality, empathy.”

The resulting guiding principles from the TC workshops were presented to all of the participants during the closing session. These impressive results ranged from written statements, poetry and images to videos. A selection of the work can be found in figure 1 (Fig. 1). A student vote was held at the end with awards given to the three groups receiving the most votes. The day’s event then ended with closing remarks by the office of academic and student affairs.

## 4. How was the project evaluated?

Since 2018 a total of 1,233 students have attended the interprofessional day and evaluated it by means of an online questionnaire using LimeSurvey. A self-devised questionnaire is used for this. In 2024 this questionnaire contained 13 items and questions about sociodemographic information. There was also the option to give written feedback. The items were rated on six-point Likert scales. The items 1. through 7. were responded to on a spectrum from “absolutely yes” (1) to “absolutely no” (6). The items 8. and 9., along with the opening session, closing session, HKI workshop and TC workshop were evaluated using Germany’s conventional scholastic grading scale ranging from “excellent” (1) to “deficient” (6).

To give an example, the evaluation results from 2024 are presented here.

The event was held on April 17, 2024. A total of 260 students participated (167 from medicine, 19 nursing and applied nursing science, 31 physiotherapy, 24 midwifery, 9 occupational/speech therapy, 10 health and health sciences) in 14 groups of 18-20 each. Of the 260 participants, 160 (61.5%) evaluated the event.

The numbers in parentheses give the mean values (M) and standard deviations (SD) for the results from 2024:

1. The event’s schedule was clearly structured (M=1.6, SD=0.72).
2. Overall, the event was well organized (M=1.6, SD=0.75).
3. I came into contact with students from other programs (M=1.9, SD=1.09).
4. I learned something new today about collaborating in interprofessional teams (MW=2.7, SD=1.37).
5. It became clear why this event could be important for me in later semesters/in my future work (M=2.0, SD=1.19).
6. The event raises my interest in continuing interprofessional contact and exchange (M=2.0, SD=0.96).
7. The event should take place again next year (M=1.7, SD=0.95).
8. I rate the opportunity to spend time in an interprofessional team as follows (M=1.8, SD=0.86).
9. Taking all of the aspects together, I assign the following final grade to interprofessional day (M=2.2, SD=0.79).

In addition to rating the Interprofessional Day overall, students were able to evaluate each of the event’s sessions and workshops separately. The highest ratings were given to the HKI workshop the students had elected to attend (M=1.8, SD=1.02). The TC workshops received an academic grade of 2.7 (SD=1.18). The opening session was evaluated on average with 3.0 (SD=1.28) and the closing session with 2.4 (SD=1.06).

The qualitative feedback provided information on which aspects of the event were successful (e.g., mixed groups, workshops, closing session) and which aspects should be changed in the future based on the students’ point of view (e.g., length of the opening session, sequence of the professions, weekday of the event).

In addition to the student evaluations, the teachers holding the workshops and those involved in the opening session are also asked for their feedback. Positive aspects were, among other things, the atmosphere in the groups, the event's organization and communications, and the idea of having an opening session. The need for improvement is seen in the (in part too small) rooms and the design of the TC workshop.

## 5. Final overall assessment and outlook

Interprofessional day at UzL offers a valuable opportunity for students from medicine and the various health professions to become acquainted with each other outside of their own degree programs, to broaden their interprofessional skills and witness the significance of collaboration in practice. The event not only promotes an understanding of each professions’ competencies, but also strengthens teamwork skills – which are indispensable for high-quality patient care.

Changes to the event’s concept will be included when planning for 2025. Cooperation with other stakeholders on campus (e.g., qualified nursing trainees and psychology students) is also being considered.

In conclusion, it is possible to assert that providing such an interprofessional impetus can be vital to developing a sense of belonging and should be expanded further in the future to sustainably promote a commitment to interprofessional collaboration and the synergies between the professions.

## Acknowledgements

We especially wish to thank all of the participants who have supported interprofessional day through their engagement and expertise. Without your efforts this project would not have been possible.

## Authors’ ORCIDs

- Annemarie Minow: [0000-0002-7836-1127]
- Janina Barth: [0000-0003-1905-1257]
- Jürgen Westermann: [0000-0001-9054-8755]

## Competing interests

The authors declare that they have no competing interests. 

## Figures and Tables

**Table 1:**
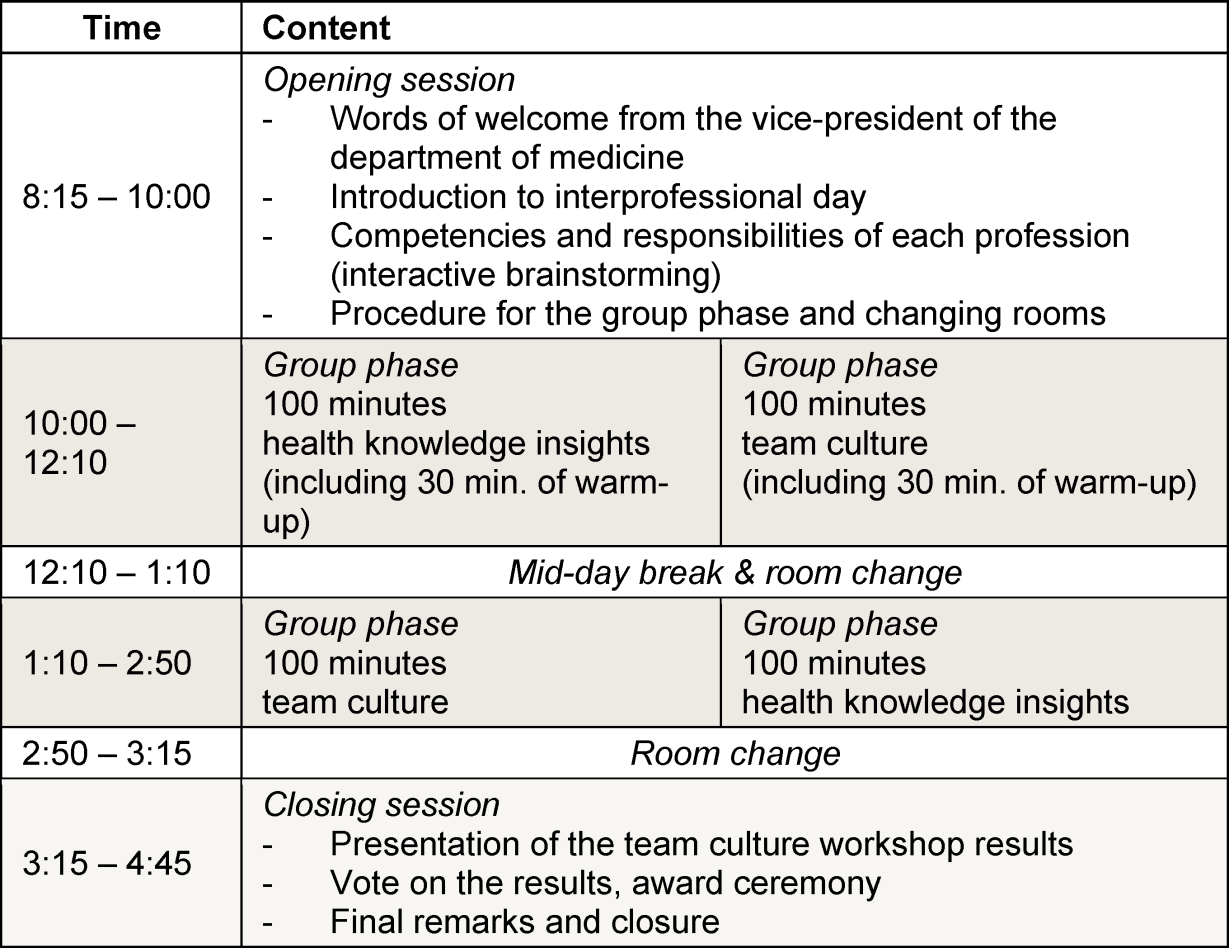
Event schedule
